# Four-Dimensionally Printed Continuous Carbon Fiber-Reinforced Shape Memory Polymer Composites with Diverse Deformation Based on an Inhomogeneous Temperature Field

**DOI:** 10.3390/polym15183740

**Published:** 2023-09-12

**Authors:** Hongyan Wang, Zhongsen Zhang, Kunkun Fu, Yan Li

**Affiliations:** 1 School of Aerospace Engineering and Applied Mechanics, Tongji University, Shanghai 200092, China; 2 Beijing Institute of Electronic System Engineering, Beijing 100854, China

**Keywords:** shape memory polymer composite, continuous carbon fiber, diverse deformation, 4D printing

## Abstract

Four-dimensionally printed continuous carbon fiber-reinforced shape memory polymer composite (CFSMPC) is a smart material with the ability to bear loads and undergo deformation. The deformation of CFSMPC can be driven by the electrothermal effect of carbon fibers. In this study, the effect of temperature on the shape memory recovery performance of polylactic acid (PLA) was first studied experimentally. Continuous carbon fibers were incorporated into PLA to design CFSMPCs with thickness gradients and hand-shaped structures, respectively. The distribution strategy of the carbon fibers was determined based on simulations of the electrically driven shape recovery process of the aforementioned structures. Both the simulations and experiments demonstrated that the electrification of the CFSMPC structures resulted in an inhomogeneous temperature field, leading to distinct deformation recovery processes. Eventually, a precise unfolding was achieved for the thickness gradient structure and the five fingers in the hand-shaped structure by utilizing a safe voltage of 6 V. This demonstrates that the 4D-printed CFSMPC with diverse deformations based on an inhomogeneous temperature field has potential applications in actuators, reconfigurable devices, and other fields.

## 1. Introduction

Since its proposal in 2013, the concept of 4D printing has been widely applied to various fields, including reconfigurable robots, biomedical equipment, aerospace, and micro clamps [1,2,3]. Controllable deformation structures can be divided into two main types based on their different deformation control mechanisms [1,2,3,4]: (1) shape memory materials, such as shape memory alloys and shape memory polymers (SMPs); and (2) double-layer structures consisting of an active material and a passive material. The active material undergoes expansion or contraction under specific external conditions, while the passive material does not exhibit significant deformation. SMPs have been extensively studied due to their advantages of a high recovery efficiency, convenient processing, and adjustability. The recovery mechanism of SMPs can be described as follows: the polymer becomes soft at temperatures above its glass transition temperature (T_g_), the polymer chains are stretched under an external force, and the chains are fixed as the temperature drops below T_g_. The storage stress of SMPs is released when they are heated up to T_g_, resulting in the restoration of the original shape on a macroscopic scale. Although shape memory effects are inherent properties of polymers, the synchronous design of materials and structural features enables more complex and controllable shape memory effects [5].

The primary manufacturing methods for SMPC are press molding [6], resin transfer molding (RTM) [7], vacuum infusion (VARI) [8], pultrusion [9], and 4D printing [10]. Among these methods, press molding, RTM, and VARI are considered conventional but complex. On the other hand, pultrusion is regarded as the most efficient manufacturing process for producing polymer composite materials. However, the emerging processing technology of 3D/4D printing has garnered significant attention due to its fast speed, low cost, simplicity, and capability of creating complex components. Four-dimensional printing allows for the creation of dynamic structures with adjustable shapes, attributes, and functionality [11,12]. Tao et al. [13] used materials with different properties to produce intelligent multi-stable metamaterials with reconfigurable, functionally deployable, mechanically adjustable, and reusable performance gradients. Nojoomi [14] utilized temperature-sensitive hydrogels to create intelligent materials with locally programmable expansion and contraction degrees and rates, inspired by spore propagation, pineal opening, and wheat distortion. Yan [15] developed wet-sensitive photosensitive polymer films (PCA, PAA, PEG) and observed that the crosslinking degree affects the humidity responsiveness of the film, thereby allowing for more accurate control of gradient pattern film deformation using humidity. Leveraging the technological advantages of 4D printing, some researchers have introduced pressure gradients in the rubber casting process to enable soft robots to achieve sequential motion through monotonic stimulation [16].

The controllable deformation structure based on a bilayer structure is achieved through the combination of two types of materials: active materials and stable materials. Commonly used active materials, such as SMP [17], photoactive polymers, liquid crystal elastomers, and hydrogels [18,19], are capable of deforming in response to external stimuli. Wang [20] utilized 3D printing to create soft claws with preprogrammed deformation, enabling them to mimic the various movements observed in plant tendrils, including bending, spiraling, and grasping. Wu [21] and Yuan [22] designed a “corrugated” deformation and an “origami crane” structure by exploiting the deformation mismatch between the elastic phase and the shape memory phase. However, these aforementioned studies rely on external environmental stimuli for activation and therefore have limited applicability.

In recent years, there has been growing interest in a new type of actuator that introduces a conductive phase into shape memory polymers (SMP) and converts electricity into heat to drive deformation. This type of actuator has found wide applications in artificial muscles, microrobots, sensors, and bionic devices [23,24]. Among these, electrothermal, electrochemical, and electrostatic layered actuators based on asymmetric extension of adjacent layers are commonly used for bending deformation. Additionally, electrothermally driven double wafer actuators exhibit reversible bending when the thermal expansion coefficients of the two contact layers do not match [25]. Lu [26,27] found that the linear response of electrical resistance to strain is mainly due to the opening/closing of the gold electrode during off-surface deformation and its expansion within the gold electrode. For carbon fiber-reinforced shape memory composites, resistance can also be used to characterize deformation. Three-dimensionally printed SMPC actuators can characterize temperature or strain through changes in resistance [28]. Wang [29] designed a bevel swing structure that changes the contact resistance upon longitudinal deformation, integrating actuation with sensing capabilities. Furthermore, a significant phenomenon exists in CFSMPC structures. The presence of carbon fiber delamination was observed [30]. Heating causes the softening of PLA, resulting in unrestricted sliding of carbon fibers. Moreover, specimen bending leads to a significant increase in carbon fiber curvature, thereby increasing the probability of fracture. These phenomena adversely affect the reproducibility of the specimens. Therefore, additional research is imperative to improve the reliability of CFSMPC structures.

While many researchers have studied the diverse deformation of shape memory materials, their focus has primarily been on the influence of various parameters on memory effects, and achieving precise control over the deformation process has rarely been explored. In the case of electrically driven SMPCs, accurate predictions of the deformation recovery process are based on the introduction of conductive materials into the SMP and observing the relationship between resistance and deformation of these materials. However, it is important to note that the deformation recovery of shape memory polymers is primarily driven by temperature changes due to phase changes. Therefore, understanding the influence of temperature on shape memory performance can provide a strong foundation for achieving controllable deformation in thermal shape memory polymers and can serve as a basis for studying complex structures and deformation mechanisms.

Shi et al. [31] conducted a finite element analysis to study the folding process of carbon nanotube shell structures reinforced by SMPC above the glass transition temperature. They also examined how the geometric parameters of the shell structure affect its flexion behavior. Baghani [32] developed a constitutive three-dimensional model for particle-reinforced SMPC, while Nishikawa et al. [33] constructed a finite element model to investigate the impact of short fibers on the shape memory properties of SMPC. Bergman and Yang [34] built a finite element beam model to simulate the shape fixation rate of continuous fiber-reinforced SMPC.

However, the existing research has not addressed the simulation of the shape memory recovery process of electrically driven shape memory composite materials. Consequently, it fails to capture the non-uniform temperature distribution and the influence of the electrothermal effect of carbon fibers on shape memory performance.

To address this gap, this study focuses on generating a heterogeneous temperature field by adjusting the regional fiber volume content through structural design. This temperature field drives the ladder deformation of the shape memory polymer. Two main structural designs are proposed: a thickness gradient structure and a hand-shaped structure. The inhomogeneity distribution of carbon fibers enables the linear distribution of the temperature field, thereby achieving the desired ladder deformation. Through thermal–electric–mechanical coupling simulations based on controllable deformation theory, the shape memory recovery mechanism under the temperature heterogeneous field is studied in depth.

## 2. Materials and Methods

### 2.1. Materials

In the present study, a continuous carbon fiber (T300-1K, Toray, Tokyo, Japan) with a linear density of 66 tex was utilized as the reinforcing material for the CFSMPC. A polylactic acid (PLA) filament with a diameter of 1.75 mm was purchased from ESUN (ePLA-Lite), Shenzhen, China.

### 2.2. Design and Preparation of CFSMPC Structures

**Figure 1:**
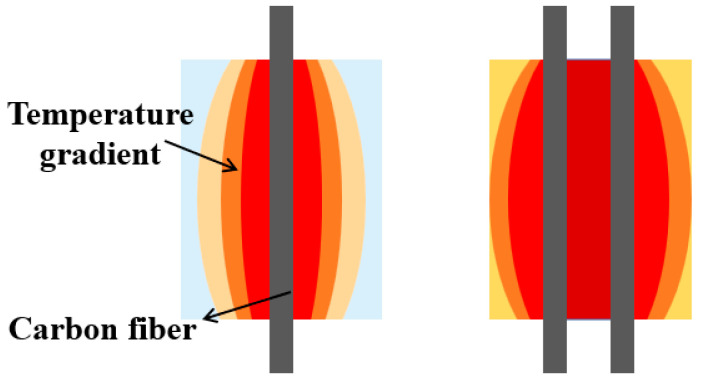
Mechanism of fiber spacing in the CFSMPC based on temperature distribution.

**Figure 2:**
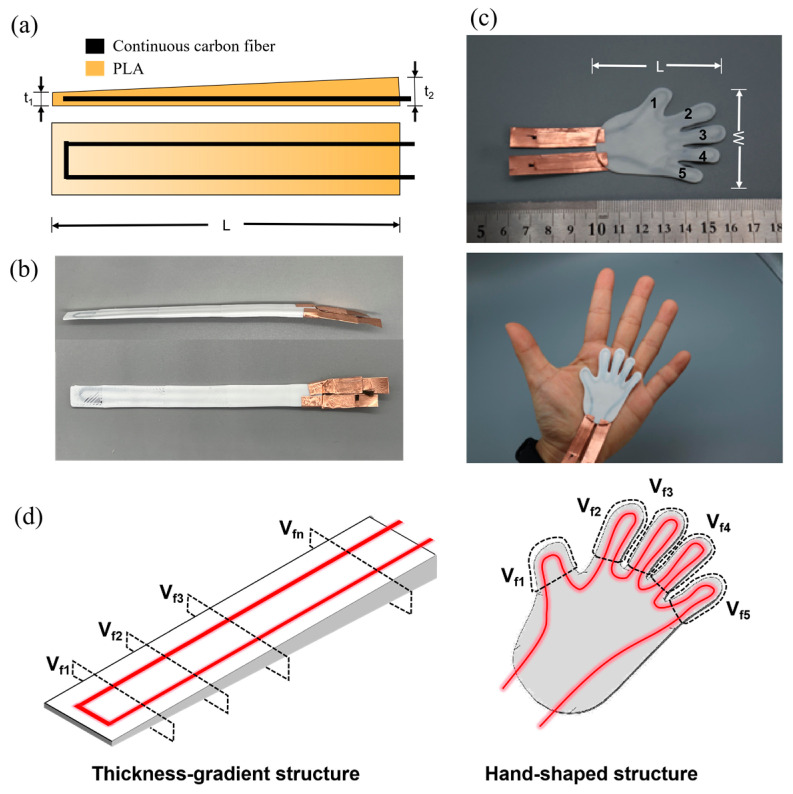
(**a**) Schematic design and (**b**) photograph of the gradient CFSMPC structure. (**c**) Photographs of the hand-like CFSMPC hand structure. (**d**) Diagrams of the regional fiber volume content for the CFSMPC structures.

The carbon fiber in the CFSMPC acted as a heat source, altering the temperature of the PLA through adjustment of the carbon fiber volume content and input voltage, consequently influencing the shape memory performance (Figure 1). Two CFSMPC structures, a thickness gradient structure and a hand-shaped structure, were designed, as shown in Figure 2. In the case of the thickness gradient structure, the gradient distribution of the regional fiber volume content was achieved by varying the longitudinal PLA thickness from t_1_ (1 mm) to t_2_ (2.5 mm), and the overall length L was 60 mm. This resulted in the PLA exhibiting a gradient temperature distribution upon energization. For the CFSMPC hand-shaped structure, the model was first designed using AutoCAD 2019 software. The overall length L was 56 mm and the width W was 48 mm. In this study, V_f_ was defined as the regional fiber volume content, as illustrated in Figure 2d. The regional fiber volume content for each finger was subsequently calculated and listed in Table 1. Three specimens were prepared for each test.

The preparation of CFSMPC involved three processes: PLA lower-sheet preparation, continuous carbon fiber printing, and PLA upper-sheet preparation. The printing path planning was performed using slice software (Cura 3.5), and the PLA samples were prepared using a 3D printer (New X1, Infinity, Taiwan, China) using the fused deposition modeling (FDM) technique. The continuous carbon fiber (CCF) was applied using an in-house 3D printer, utilizing an in situ impregnation technique detailed in a prior study [35]. The specific printing parameters are listed in Table 2.

### 2.3. Electrically Driven Shape Memory Test

#### 2.3.1. Test Methods

**Figure 3:**
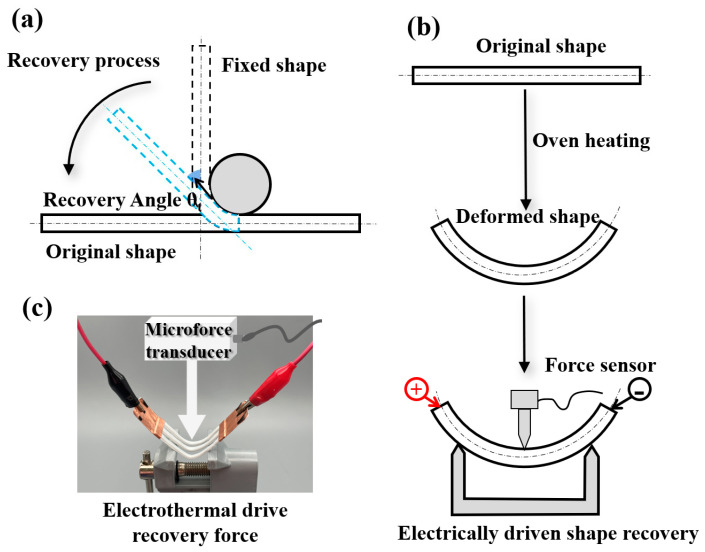
(**a**) Schematic diagram of a shape memory cycle. (**b**) Recovery force test device during electrically driven shape memory recovery. (**c**) Photograph of electrically driven shape memory test fixture.

The shape memory polymers went through a two-step shape memory cycle involving deformation and recovery. The deformation process is depicted in Figure 3a. Initially, the sample was heated to T_g_ = 67 °C using a hot air gun and formed into a temporary shape by bending. It was then kept at room temperature until it cooled down, resulting in fixation of the temporary shape. The recovery process is illustrated in Figure 3b, where the shape of the sample gradually returns to its initial form as the PLA temperature reaches T_g_. High-definition cameras recorded the shape recovery process of the sample and captured images of the sample’s shape at various time intervals. An infrared thermal imager recorded the temperature distribution during the experiment. Figure 3c showcases the experimental equipment used to measure the electrothermal deformation force. A microforce sensor (DYLY-109) with a maximum force capacity of 500 mN from Dayang Sensor Technology Company, Shenzhen, China was utilized to measure the recovery force. The ambient temperature was controlled by air conditioning at 25 °C, whereas the temperature of the specimen approached approximately 22 °C.

#### 2.3.2. Electrically Driven Shape Memory Test of the Thickness Gradient Structure

For the unfold test, the specimen was heated using a heat gun until it reached the glass transition temperature. Subsequently, a cylinder with a diameter of 30 mm was utilized as a mold to bend the specimen around it. Once the desired shape was achieved, the specimen was allowed to cool down to room temperature, accounting for minor spring-back effects. This process resulted in the specimen achieving a nearly circular temporary shape. The thicker end of the gradient structure remained fixed, while the electrodes were clamped at both ends of the U-shaped carbon fiber. Upon energization, the specimen gradually unfolded due to the electrothermal action of the carbon fiber, ultimately returning to its initial shape.

For the grasp test, a heat gun was employed to heat the gradient structure, facilitating the softening of the specimen. The softened specimen was then wrapped around a glass rod and cooled to room temperature while maintaining this position. As a result, the fixed shape of the specimen effectively locked to the glass rod. Subsequently, one end of the specimen was fixed. The specimen progressively unfolded when voltage was applied to both ends of the carbon fibers. A camera was utilized to record the recovery response of the specimen.

#### 2.3.3. Electrically Driven Shape Memory Test of the Hand-like Structure

For the unfold test, the palm was heated using a heat gun until it reached the glass transition temperature. Once softened, the fingers were bent and the palm was fixed until it cooled down, allowing for the temporary shape to be set.

For the grip test, to further investigate the functionalization application of CFSMPC structures, the hand-like gripper structure was utilized for lifting and releasing heavy objects. The basket containing the heavy objects was placed within the palm of the gripper structure. By clamping the palm and energizing the carbon fiber, the process of releasing heavy objects was realized.

### 2.4. Finite Element Modeling

**Figure 4:**
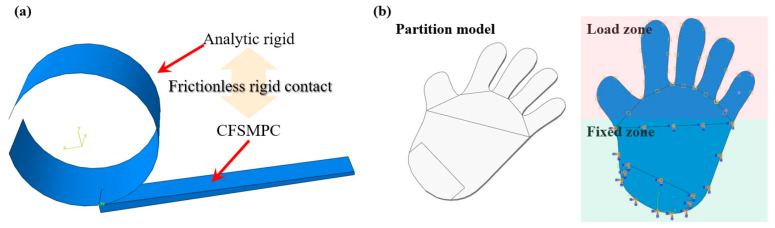
(**a**) Finite element model of the gradient CFSMPC structure. (**b**) Finite element model of the hand-shaped CFSMPC structure.

In order to achieve active control of the deformation, a thermal–electric–mechanical coupling numerical model was established, as shown in Figure 4a. A transiently coupled thermal–electrical step was carried out using ABAQUS 2019/Standard for the analysis. The carbon fibers were meshed using eight-node linear-coupled thermal–electrical brick (DC3D4) elements. On the other hand, the PLA matrix was meshed using four-node linear heat transfer tetrahedron (DC3D8E) elements. The material parameters for the PLA and carbon fibers used in the finite element models are listed in Table 3. The simulation processes for the shape memory recovery were as follows:

Step 1, apply a uniform temperature field and apply an external force to pre-deform the CFSMPC;

Step 2, maintain the external force while reducing the temperature to fix the deformation of the CFSMPC;

Step 3, keep the temperature low and remove the external force;

Step 4, electrify the carbon fiber and drive the PLA through the carbon fiber electro-heating effect. 

The load in Abaqus was defined using the LOAD module. A current of 83 A/mm^2^ was specified at the end of the carbon fiber. The boundary conditions included an electric field and a temperature field, with an initial temperature of 22 °C for the entire specimen and zero potential set at the other end of the carbon fiber. Therefore, a closed loop was formed.

Figure 4b depicts the hand-shaped CFSMPC finite element geometry model that was partitioned to set the constraints. The palm part served as a fixed constraint, while the external force for Step 1 to Step 2 was applied to the finger area. For simplification, the carbon fibers were considered as 0.2 mm diameter columns in this study. Additionally, a viscoelastic material parameter was assigned to PLA to simulate the viscoelastic deformation.

## 3. Results and Discussion

### 3.1. Effect of Temperature on the Shape Memory Performance of the PLA

The shape memory performance of PLA is closely linked to temperature; therefore, the PLA shape memory experiment was conducted within a specific temperature range. The specimen was heated and maintained at a constant temperature in a temperature chamber until it reached a steady state. When the temperature exceeded T_g_, the PLA underwent a softening process and gradually returned to its original state. Consequently, the variable considered in this study was the steady-state temperature of the temperature chamber, which was then used to analyze the correlation between the shape memory performance of PLA and this variable.

**Figure 5:**
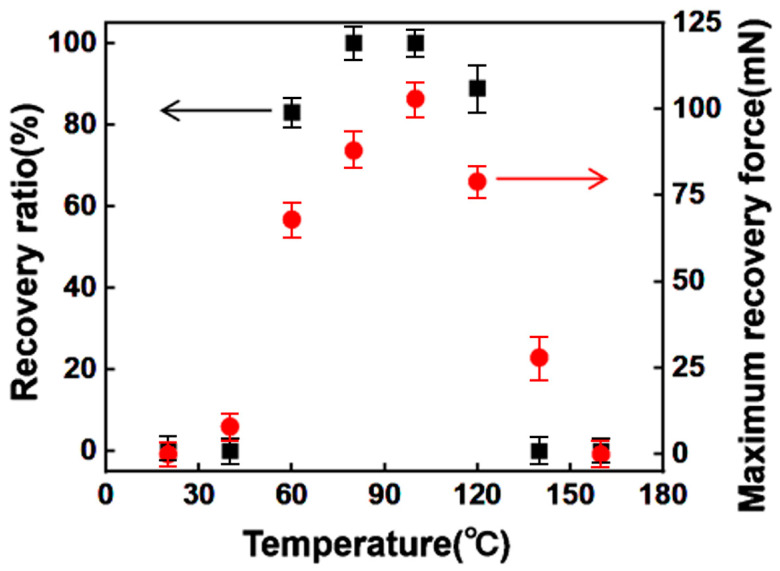
The recovery ratio and maximum recovery force at different PLA temperatures.

The relationship between the measured recovery ratio/maximum recovery force and the temperature is presented in Figure 5. It demonstrates that when the temperature is below T_g_, the neat PLA is in a glass state, resulting in a maximum recovery force of only 0.5 mN. As the temperature gradually increases, the reversible phase of the PLA begins to change, causing the material to become softer. However, when the temperature reaches 97 °C, the recovery force undergoes a significant decline. To accurately characterize the temperature range suitable for PLA, the interval of 67–110 °C was identified as the effective temperature zone for achieving highly efficient recovery. It is recommended to maintain the PLA within this temperature range in order to obtain an optimal recovery force.

### 3.2. Shape Memory Performance Electrically Driven by the Thickness Gradient Structure

#### 3.2.1. The Thermal Temperature Field of the CFSMPC Thickness Gradient Structure

**Figure 6:**
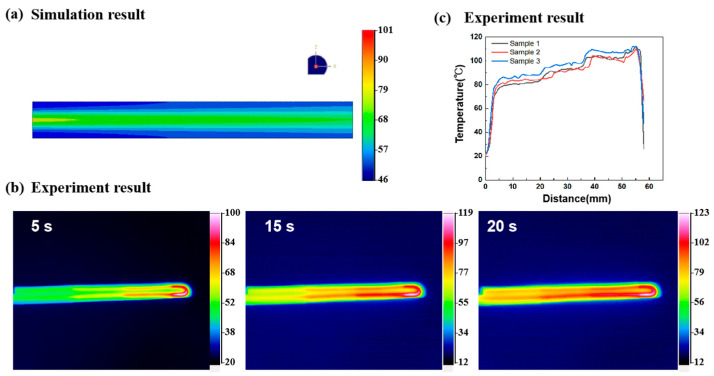
(**a**) The simulation results of the temperature distribution with an energization of 20 s. (**b**) Infrared thermal images, (**c**) temperature distribution for the electrically stimulated CFSMPC gradient structure after a 20 s duration.

By applying a surface current load to the carbon fiber, the PLA was driven by the electrothermal effect of the carbon fiber. Figure 6a illustrates the emulated electrothermal temperature distribution in the CFSMPC gradient structure under a voltage of 6 V. It is evident that there was a linear change in temperature in the axial direction, with the end of thickness t_1_ = 1 mm having the highest temperature of 100.5 °C, and the end of thickness t_2_ = 2.5 mm having the lowest temperature of 46.2 °C. The temperature decreased from the carbon fiber to the PLA.

Figure 6b depicts the temperature distribution of the sample after being powered on for 5 s, 15 s, and 20 s at a voltage of 6 V. As the power-on time increased, the sample temperature gradually increased while still maintaining an axial temperature gradient. Figure 6c corresponds to the temperature distribution in the lengthwise direction under the experimental conditions at 6 V, illustrating a significant temperature gradient caused by the difference in the regional fiber volume content. The lowest temperature recorded was 80 °C, while the highest temperature was 107 °C; both within the effective temperature range.

#### 3.2.2. The Shape Recovery Process of the CFSMPC Thickness Gradient Structure

**Figure 7:**
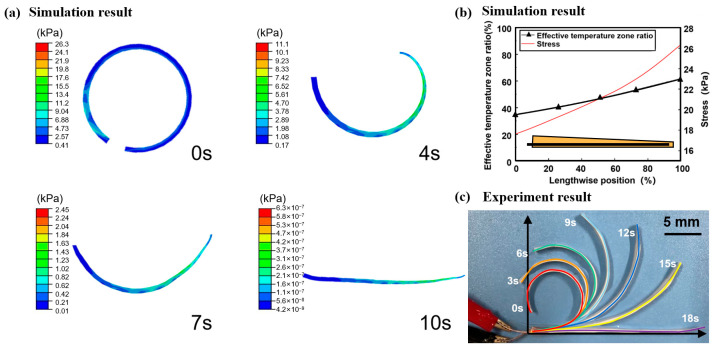
(**a**) Simulation result of the recovery process of the CFSMPC gradient structure under a driving voltage of 6 V. (**b**) Simulated effective temperature zone ratio and stress along the lengthwise position for the CFSMPC gradient structure. (**c**) Experimental results of the recovery process of the CFSMPC gradient structure.

Figure 7a depicts a stress cloud diagram of the CFSMPC gradient structure during the recovery process. At the beginning of the process (0 s), the gradient CFSMPC structure completed pre-deformation, resulting in storage stress within the structure. The maximum stress observed was 26 kPa, with the axial upward stress decreasing from the thin end to the thick end. After 10 s, the entire structure achieved the desired shape recovery. This indicates that the shape memory performance was directly influenced by the internal stress, and the design of the gradient structure can effectively achieve a controllable step deformation.

Following the deformation of the gradient structure, the CFSMPC exhibited reserved stress. Figure 7b displays the maximum axial stress distribution of the gradient structure, showing a similar trend to the effective temperature area ratio. As the structure thickness decreased, the proportion of the effective temperature area gradually increased, leading to a gradual increase in the storage stress within the corresponding area. This can be attributed to the regional increase in the regional fiber volume content as the thickness decreased, resulting in elevated storage stress after pre-deformation. When electrified, the carbon fiber’s Joule-heated power remained consistent across all the areas. Consequently, the PLA temperature was higher in regions with a higher regional fiber volume content, highlighting the greater proportion of the effective temperature area.

Figure 7c reveals that the curvature changed most rapidly at the end with a high regional fiber volume content, while the curvature changed more slowly in the sections with a low regional fiber volume content. This discrepancy arises from the PLA in the thicker end mainly performing within a low-temperature zone. Consequently, the PLA deformation and recovery within the effective temperature zone were inhibited, leading to a slower recovery speed. This experiment illustrates that the introduction of inhomogeneous temperature fields through structural design enhances the diversity of electrically driven CFSMPC deformation.

#### 3.2.3. Grasp Demonstration of the CFSMPC Thickness Gradient Structure

**Figure 8:**
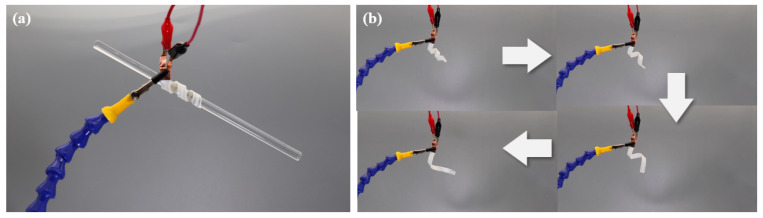
(**a**) Grasping of a glass bar. (**b**) Recovery progress of the CFSMPC gradient structure.

In order to verify the load-bearing capability of the CFSMPC gradient structure, a clamp (depicted in Figure 8a) was prepared with a mass of 30 g. This clamp demonstrated a satisfactory bearing capacity. It is noteworthy that the clamp holder successfully grasped and lifted a 200 g glass bar, resulting in a weight ratio of 6.8:1. Furthermore, upon electrification, the fixed gradient structure featuring a helix with a uniform pitch was automatically untwisted to attain intricate deformation (Figure 8b).

### 3.3. Shape Memory Performance of the Electrically Driven CFSMPC Hand-Shaped Structure

#### 3.3.1. Simulation Results

**Figure 9:**
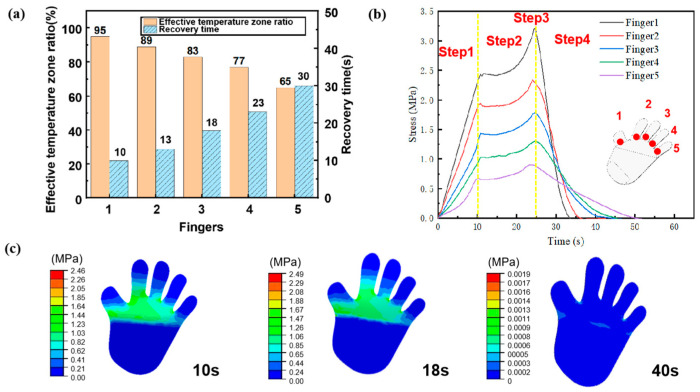
(**a**) Effective temperature zone and recovery time for each finger. (**b**) Stress variation during the electrically driven recovery process. (**c**) Stress distribution at different recovery steps.

Figure 9a displays the relationship between the effective temperature interval, recovery time, and the final steady-state temperature distribution of each finger. The hand CFSMPC could fully recover within 30 s under the electric drive. The recovery speeds of the five fingers varied, with values of 10 s, 13 s, 18 s, 23 s, and 30 s. This was attributed to the varying regional fiber volume content, with values of 0.95%, 0.8%, 0.75%, 0.65%, and 0.6%, respectively. A higher regional carbon fiber volume content generates more Joules of heat simultaneously, leading to increased heat conduction to the PLA and consequently achieving a faster recovery.

Figure 9b illustrates the stress simulation results of the electrically driven recovery process of the hand-like CFSMPC gripper. The stress in the structure was mainly concentrated at the root of the finger. This is because the bending deformation primarily occured at the root and fingertips. However, the palm showed almost no deformation, resulting in no internal stress. Figure 9c illustrates the stress variation process at the bending center of each finger. Prior to the initiation of the electrically driven recovery, the storage stress within each finger decreased from finger 1 to 5 as a result of varying PLA areas undergoing temporary deformation. When a larger amount of PLA bent, more energy was stored in the structure, resulting in higher internal stress after cooling and fixation. After being electrified, the stored stress was gradually released, with the full release sequence of stress in the five fingers occurring from Finger 1 to 5.

#### 3.3.2. Experimental Results

**Figure 10:**
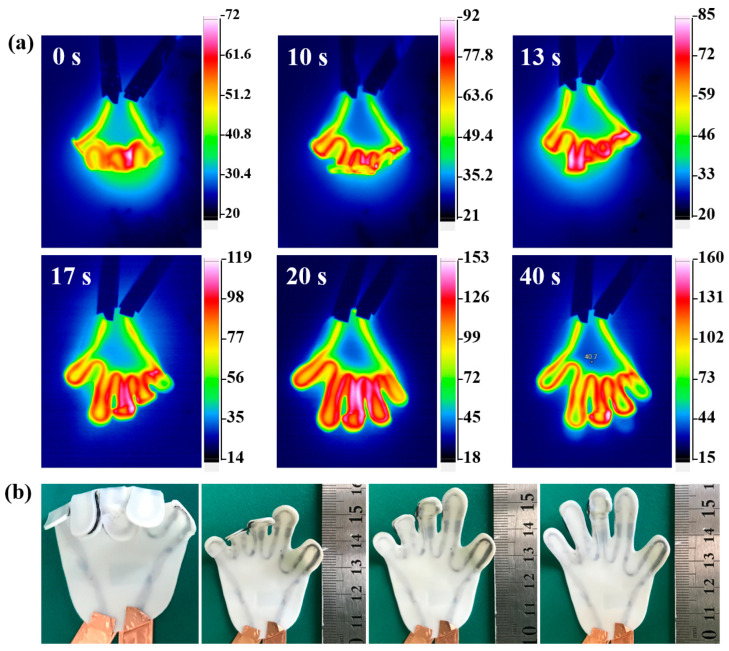
(**a**) Infrared thermal images and (**b**) photographs of the hand-like CFSMPC gripper during the electrically driven recovery process.

The initial state of the hand CFSMPC was flat and spread. Then, the sample was heated using the hot air gun, and the temperature was kept at 100 °C. Next, the deformation was applied. The external force fixed the sample naturally after cooling. A 6 V driving voltage was connected at both ends of the carbon fiber. Then, t hand was gradually heated and began to recover. Figure 10a shows the heating process of CFSMPC at a 6 V driving voltage. It can be seen that a different temperature distribution was presented due to the different regional fiber volume content of each finger. Figure 10b shows the hand unfolding process, with the fingers transforming from 1 to 5. As shown in the infrared diagram, the temperature of the fingers gradually increased with time, and the temperature area of different fingers also varied. In the end, all five fingers responded completely.

The hand-like CFSMPC grip initially had a flat and spread state. It was then subjected to heating using a hot air gun, with the temperature maintained at 100 °C. Subsequently, deformation was applied, which would naturally fix the sample upon cooling. A 6 V driving voltage was connected to both ends of the carbon fiber. As a result, the hand gradually heated up and began to recover. Figure 10a illustrates the heating process of the CFSMPC at a 6 V driving voltage, revealing that different temperature distributions were observed due to the varying regional fiber volume content in each finger. Figure 10b displays the unfolding process of the hand, with the fingers transitioning from 1 to 5. The infrared thermal images demonstrate that the temperature of the fingers increased gradually over time, and the temperature areas of the different fingers also varied. Finally, all five fingers exhibited a complete recovery.

#### 3.3.3. Grip Demonstration of the Hand-like CFSMPC Hand-Shaped Structure

**Figure 11:**
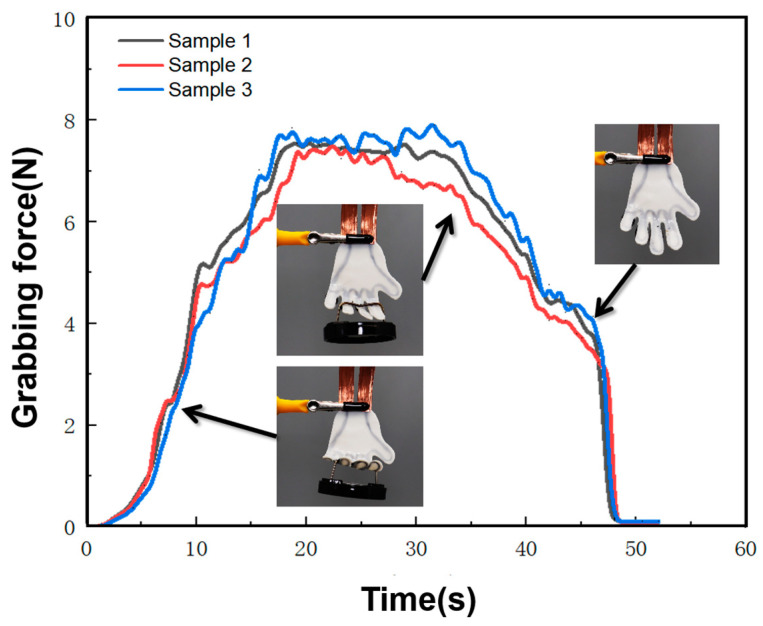
Grabbing force–time curve for weight lifting and releasing of the hand-like CFSMPC gripper.

To investigate the functionalization of the hand-like CFSMPC gripper structure, a heavy object was grabbed and lifted, as shown in Figure 11. The hand itself weighed 30 g, while the weight of the object was 780 g. Upon being energized, the hand underwent transformation and recovery, allowing for the release of the heavy object within 18 s. This versatile structure offers numerous advantages, such as its suitability for use in diverse remote environments, as well as its ability to achieve various gripping and hanging capabilities.

## 4. Conclusions

In this study, novel structures of CFSMPC were designed to investigate the feasibility of diverse deformation mechanisms. By employing a gradient distribution of carbon fibers, an electric current-induced gradient temperature distribution was observed in the polylactic acid (PLA), resulting in noticeable gradient deformation effects. The CFSMPC gradient structure exhibited a spiral-like deformation recovery within a single electrical stimulation cycle, with a recovery time of 18 s. Furthermore, a hand-like CFSMPC gripper structure was developed, which demonstrated sequential finger unfolding under low electrical stimulation, achieving complete recovery within 43 s. This study presents a cost-effective and operationally convenient method. The current experimental results illustrate that the non-uniform temperature field has the potential to cause complex deformations, thereby laying the groundwork for accurate control of deformations through global temperature distribution. The primary objective of future research is to attain more precise control. The structure was designed based on the anticipated degree of deformation, with the potential for reaching magnitudes as high as °/mm.

## Figures and Tables

**Table 1:** Structural parameters for the “five fingers” of the CFSMPC gripper.

Finger No.	1	2	3	4	5
Finger area (mm^2^)	420	390	370	320	280
Carbon fiber bundle length (mm)	39.9	31.2	27.75	20.8	16.8
Regional fiber volume content (%)	0.95	0.8	0.75	0.65	0.6

**Table 2:** 3D Printing parameters of the CFSMPC structures.

Print Parameters	Materials	
	PLA	CCF/PLA
Line width (mm)	0.4	as required
Nozzle diameter (mm)	0.4	0.8
Layer thickness (mm)	0.2	0.2
Print temperature (°C)	220	220
Print speed (mm/s)	40	5

**Table 3:** Material parameters used for the finite element simulation.

Parameters	Materials	
	CF	PLA
Density (g/cm^3^)	1.76	1.24
Specific heat (J/(kg °C))	700	200
Conductivity (W/(mm °C))	6000	250
Joule heat fraction	1	-
Viscoelastic properties (WLF equation)	-	C_1_ = 15, C_2_ = 67

## Data Availability

The data are available upon request due to privacy restrictions.
